# Diagnostic Accuracy and Clinical Impact of Sentinel Lymph Node Sampling in Endometrial Cancer at High Risk of Recurrence: A Meta-Analysis

**DOI:** 10.3390/jcm9123874

**Published:** 2020-11-28

**Authors:** Lise Lecointre, Massimo Lodi, Émilie Faller, Thomas Boisramé, Vincent Agnus, Jean-Jacques Baldauf, Benoît Gallix, Chérif Akladios

**Affiliations:** 1 Department of Gynecologic Surgery, Hôpitaux Universitaires de Strasbourg, 67200 Strasbourg, France; lise.lecointre@chru-strasbourg.fr (L.L.); emilie.faller@chru-strasbourg.fr (É.F.); thomas.boisrame@chru-strasbourg.fr (T.B.); jean-jaques.baldauf@chru-strasbourg.fr (J.-J.B.); cherif.akladios@chru-strasbourg.fr (C.A.); 2 I-Cube UMR 7357-Laboratoire des Sciences de L’ingénieur, de L’informatique et de L’imagerie, Université de Strasbourg, 67081 Strasbourg, France; benoit.gallix@ihu-strasbourg.fr; 3 Institut hospitalo-universitaire (IHU), Institute for Minimally Invasive Hybrid Image-Guided Surgery, Université de Strasbourg, 67081 Strasbourg, France; vincent.agnus@ihu-strasbourg.fr; 4 Functional Genomics and Cancer Departement, Institut de Génétique et de Biologie Moléculaire et Cellulaire (IGBMC), INSERM U1258, CNRS UMR7104, 67400 llkirch-Graffenstaden, France; 5 Department of Diagnostic Radiology, McGill University, Montreal, QC H3A 2T5, Canada

**Keywords:** endometrial cancer, sentinel lymph node, high risk

## Abstract

Purpose. To assess the value of sentinel lymph node (SLN) sampling in high risk endometrial cancer according to the ESMO-ESGO-ESTRO classification. Methods. We performed a comprehensive search on PubMed for clinical trials evaluating SLN sampling in patients with high risk endometrial cancer: stage I endometrioid, grade 3, with at least 50% myometrial invasion, regardless of lymphovascular space invasion status; or stage II; or node-negative stage III endometrioid, no residual disease; or non-endometrioid (serous or clear cell or undifferentiated carcinoma, or carcinosarcoma). All patients underwent SLN sampling followed by pelvic with or without para-aortic lymphadenectomy. Results. We included 17 original studies concerning 1322 women. Mean detection rates were 89% for unilateral and 68% for bilateral. Pooled sensitivity was 88.5% (95%CI: 81.2–93.2%), negative predictive value was 96.0% (95%CI: 93.1–97.7%), and false negative rate was 11.5% (95%CI: 6.8; 18.8%). We noted heterogeneity in SLN techniques between studies, concerning the tracer and its detection, the injection site, the number of injections, and the surgical approach. Finally, we found a correlation between the number of patients included and the SLN sampling performances. Discussion. This meta-analysis estimated the SLN sampling performances in high risk endometrial cancer patients. Data from the literature show the feasibility, the safety, the limits, and the impact on surgical de-escalation of this technique. In conclusion, our study supports the hypothesis that SLN sampling could be a valuable technique to diagnose lymph node involvement for patients with high risk endometrial cancer in replacement of conventional lymphadenectomy. Consequently, randomized clinical trials are necessary to confirm this hypothesis.

## 1. Introduction

Endometrial cancer represents the sixth diagnosed cancer among women, representing 382,069 new cases and 89,929 deaths in 2018 worldwide [1]. This cancer usually affects women after menopause, with the average age of patients at diagnosis being 68 years. It therefore affects an elderly population, sometimes with serious comorbidities that must be taken into account in surgical management. Approximately 80% of new cases are diagnosed at the FIGO (Iternational Federation of Gynecology and Obstetrics) stage I [2,3,4], but it is a heterogeneous pathology and 5-year overall survival can vary from 92% to 42%, depending on its histological characteristics [5]. Recently, a consensus conference including three different European learned societies (ESMO-ESGO-ESTRO) established new recommendations in order to standardize the management of early stage endometrial cancer and one of the main points is the emergence of a new subgroup of patients at risk of recurrence (high-intermediate risk group) to better define the indications for initial treatment [2].

Surgery is the mainstay of treatment for early-stage endometrial cancer [2,3,4], but over the last decades, concepts about surgery have evolved and more specifically, that of lymph node staging. On the one hand, hysterectomy with adnexectomy allows the removal of the tumor and its histological classification, including subtype, grade, and depth myometrial invasion. On the other hand, the assessment and estimation of lymph node status, using Magnetic Resonance Imaging, Positron Emission Tomography or Computed Tomography, and preoperative biopsy criteria, is still debated in the international literature [6,7,8]. Management of stage I endometrial cancer has been marked by therapeutic de-escalation, particularly with regard to the performance of lymphadenectomy. Indeed, several randomized studies have demonstrated the absence of benefits in terms of long-term survival and recurrence-free survival from performing systematic lymphadenectomy [9] in stage I endometrial cancers, especially since it causes morbidity and long-term complications, such as lymphoceles and lymphoedema [10,11,12,13].

However, the rationale for adjuvant therapeutics management of endometrial cancer is based on the estimation of a theoretical risk of recurrence and lymph node metastasis. For that reason, the concept of sentinel lymph node biopsy (SLN) has been proposed as an alternative to conventional lymphadenectomy while avoiding the total absence of lymph node sampling [14]. Of interest, sentinel lymph node sampling makes pathological ultrastaging accessible and therefore, the detection of low volume disease not detectable by conventional histological examination is executed in the case of extensive lymphadenectomy [15,16].

The challenge is not to overtreat patients at low risk of recurrence and, conversely, not to underestimate the risk of recurrence [2,3,4]. French recommendations recommend that pelvic SLN is recommended in early-stage endometrial cancers at low and intermediate risk [4]. As the clinical impact of the SLN procedure has not been proven, the European societies (ESMO-ESGO-ESTRO) consider it to be an experimental procedure and do not recommend it [2]. On the other hand, in the latest American recommendations (NCCN), pelvic SLN is recommended pre-operatively for low risks, intermediate risks, and high risks of recurrence, while remaining associated with lombo-aortic lymphadenectomy pending additional data [3].

Although accumulated data underline the safety and effectiveness of SLN biopsy, only few studies, comparing SLN biopsy vs. lymphadenectomy, are still available in endometrial cancer at high risk of recurrence. We aimed to assess and review the value of SLN sampling in high risk endometrial cancer according to the ESMO-ESGO-ESTRO classification in comparison to conventional lymphadenectomy.

## 2. Patients and Methods

### 2.1. Bibliographic Selection

**Figure 1:**
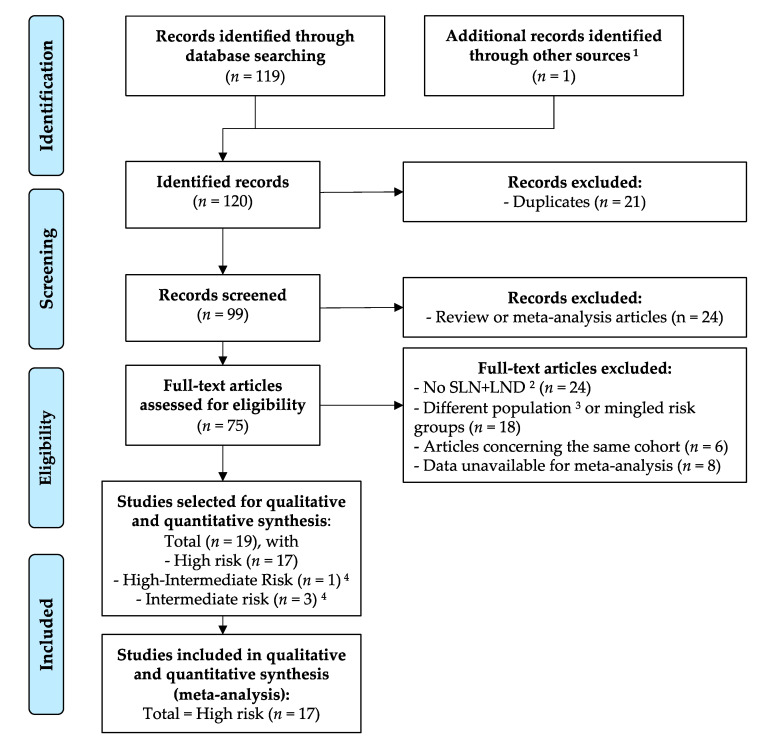
Bibliographic selection flow diagram according to the PRISMA statement. ^1^ The other source was the bibliography of a review article on the topic. ^2^ Sentinel lymph node (SLN) procedure followed by lymph-node dissection (LND). ^3^ Not high, high-intermediate, or intermediate risk groups. ^4^ Intermediate and high-intermediate risk groups were excluded from the analysis because there were too few studies to analyze. Some articles reported data for different risk groups; consequently, the sum of each risk group is above the total selected articles.

We followed PRISMA guidelines for this systematic review and meta-analysis [17]. An initial PubMed (MEDLINE) search based on the following terms: ‘‘endometrial cancer” and “sentinel lymph node” and “high risk” gave 119 entries. After duplicates were removed (*n* = 20), the selection of articles of interest was made by two independent and blinded reviewers (L.L. and M.L.). Studies were excluded if: (1) they were meta-analyses, correspondence, literature reviews, or fundamental research articles (*n* = 24); (2) they had a non-direct relationship with the subject being studied (*n* = 24); (3) inclusion criteria did not correspond with the ESMO-ESGO-ESTRO high risk groups (*n* = 18); or (4) because data were not available for meta-analysis (*n* = 8). Further research through the bibliographies of selected articles and certain review articles allowed us to single out 1 additional article that was considered relevant to the study. Then, 17 studies related to the high risk group were chosen for qualitative and quantitative meta-analysis. The bibliographic selection is summarized in Figure 1, according to the PRISMA statement [17].

### 2.2. Inclusion Criteria

All patients with high risk endometrial cancer according to the ESMO-ESGO-ESTRO Consensus Conference [2] were included: stage I endometrioid, grade 3, with at least 50% myometrial invasion, regardless of lymphovascular space invasion status; or stage II; or node-negative stage III endometrioid (IIIa and IIIb), no residual disease; or non-endometrioid (serous or clear cell or undifferentiated carcinoma, or carcinosarcoma).

### 2.3. Data Extraction

Working independently, two reviewers (L.L. and M.L.) extracted the data using a piloted and standardized form. The following information was extracted: study design variables, patients’ characteristics, surgical details of procedure, and surgery-related outcomes. No discordance was noted during the data extraction.

### 2.4. Outcomes

From each article, we retrieved clinical and pathological data and measures of the performance of the sentinel lymph node (SLN) sampling followed by pelvic ± para-aortic lymphadenectomy: true and false-negative and positive patients, sensitivity, specificity, positive and negative predictive value, etc. If available, we also included survival data (overall and disease-free survival, respectively, OS and DFS) and surgical complications (intra- and post-operative.

### 2.5. Statistical Analysis

Statistical analysis was performed with R version 3.5.1 (2018-07-02) [18] and packages *meta* [19] and *mada* [20]. We calculated the pooled sensitivity and negative predictive value (NPV) with a random intercept logistic regression model and logit transformation. We quantified heterogeneity with a maximum-likelihood estimator for tau^2^ and calculated the Higgins’ I^2^ statistic. For the test of heterogeneity, the Cochran Q *p*-value was obtained with the Wald-type test. For individual studies, we used the Clopper-Pearson confidence interval method. As false positive results of this technique were not expected, we applied a continuity correction of 0.5 in studies with zero cell frequencies, but only to calculate individual study results. For correlation, we used the Pearson method.

## 3. Results

For this meta-analysis, we included 17 articles [21,22,23,24,25,26,27,28,29,30,31,32,33,34,35,36,37] concerning 1322 women with high risk endometrial cancer. All women underwent the SLN procedure followed by pelvic with or without para-aortic lymphadenectomy.

### 3.1. SLN Techniques

We observed a certain degree of heterogeneity concerning SLN techniques that were used, whether it was the tracer and its detection, the injection site, or the number of injections, and the surgical approach. Data about SLN techniques are reported in Table 1.

The most common tracers were blue dye (methylene or isosulfane) in 11 studies (64.7%), indocyanine green in 11 studies (64.7%), and radioactive nanocolloid (^99m^Technecium) in six studies (35.3%). Those tracers were used alone (nine studies, 52.9%) or in combination (eight studies, 47.1%).

Injection site, when specified (16 studies, 94.1%), was cervical (12 studies, 70.6%) or sub-serosal myometrium (four studies, 23.5%). The number of injections varied from 1 to 4 and in some studies, reinjection was performed during the intervention.

Surgical approach was mainly laparoscopy, whether conventional (nine studies, 52.9%) or robotic assisted (seven studies, 41.1%), however some studies have also reported laparotomies (four studies, 23.5%). In three studies, the surgical approach was not specified. Surgical approach was homogenous (i.e., only one per study) in 10 studies, while four reported at least two different surgical approaches.

### 3.2. Detection Rates

As reported in Table 1, the unilateral detection rate was available in 15 studies and ranged from 67% [23] to 100% [30,31], with a mean value of 89%. The bilateral detection rate was reported in 12 articles and ranged from 41% [26] to 95% [31], with a mean value of 68%.

### 3.3. Global Measures of SLN Performance

**Figure 2:**
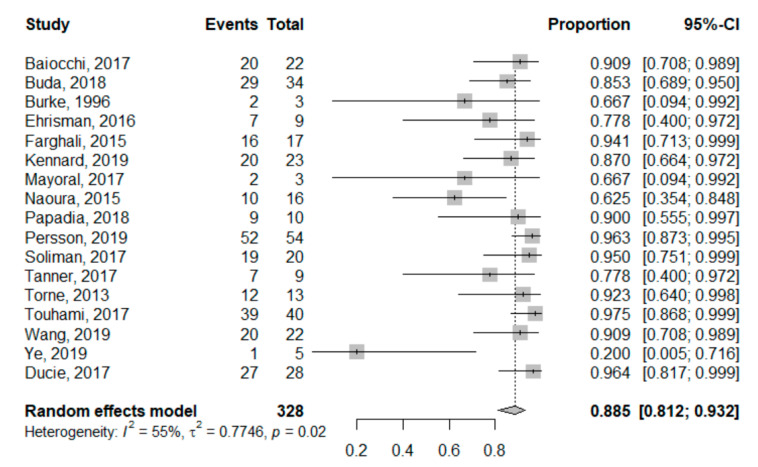
Univariate pooled sensitivity analysis. Forest plot reporting the univariate analysis of individual and pooled sensitivity. The Events column corresponds to the True Positive cases, while the Total is all node-positive patients (True Positives + False Negatives).

SLN sensitivity ranged from 20% [37] to 97.5% [35]. The pooled global sensitivity was 88.5% (95%CI: 81.2–93.2%). Sensitivity for each study is reported in the forest plot corresponding to Figure 2. Heterogeneity for this analysis was moderate as the Higgins’ I^2^ index was 55% (*p* = 0.02).

**Figure 3:**
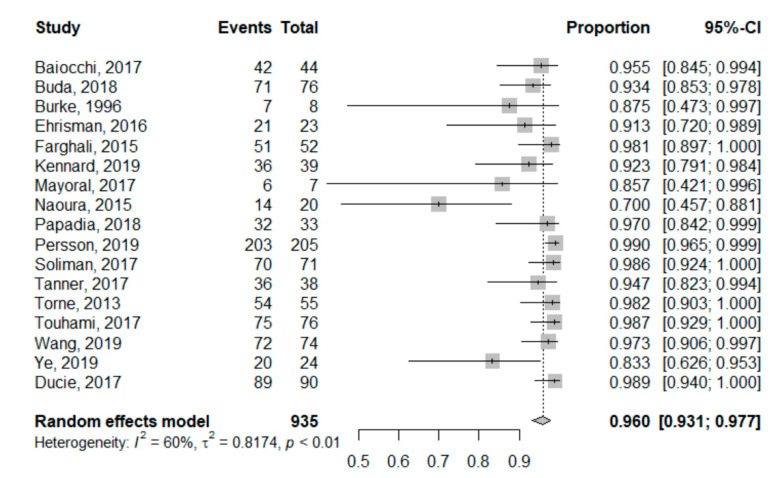
Univariate pooled negative predictive value analysis. Forest plot reporting the univariate analysis of individual and pooled negative predictive value. The Events column corresponds to the True Negative cases, while the Total is all SLN-negative patients (True Negatives + False Negatives).

Concerning NPV, we found that it ranged from 70% [28] to 99% [31]. The pooled global NPV was 96.0% (95%CI: 93.1–97.7%). NPV for each study is reported in the forest plot corresponding to Figure 3. Heterogeneity for this second analysis was higher, although still moderate, as the Higgins’ I^2^ index was 60% (*p* < 0.01).

**Figure 4:**
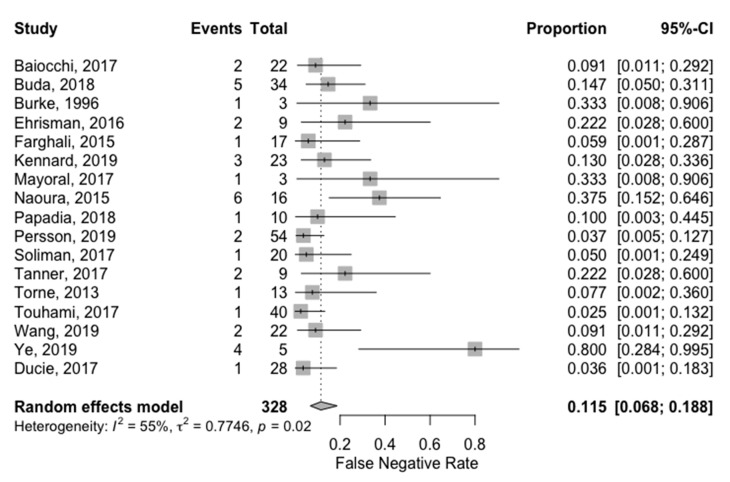
Univariate pooled false negative rate analysis. Forest plot reporting the univariate analysis of individual and pooled false negative rate. The Events column corresponds to the False Negative cases, while the Total is all node-positive patients (False Negatives + True Positives).

Global False Negative Rate (FNR) ranged from 2.5% [35] to 80% [37]. The pooled FNR was 11.5% (95%CI: 6.8%; 18.8%). FNR for each study is reported in the forest plot corresponding to Figure 4. Heterogeneity for this analysis was moderate as the Higgins’ I^2^ index was 55% (*p* = 0.015).

**Figure 5:**
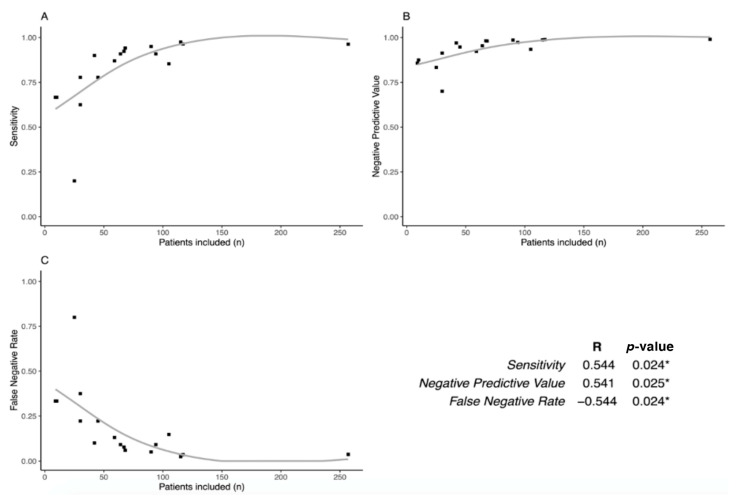
Learning curve of the SLN technique. Plots comparing Sensitivity (**a**), Negative Predictive Value (**b**), and False Negative Rate **(c)** to the number of patients included for each study. The table shows the correlation between these performances and the number of patients: Sensitivity and Negative Predictive Value are correlated (*p* = 0.024 and *p* = 0.025, respectively), while False Negative Rate is inversely correlated with the number of patients included (*p* = 0.024). * means a significative *p*-value (< 0.05).

Then, we studied if SLN performances were associated with the population size of each study (Figure 5). Interestingly, we found that sensitivity and NPV are positively correlated with the number of patients included (*p* = 0.024 and *p* = 0.025, respectively), while FNR is inversely correlated (*p* = 0.024).

We further investigated if there was a difference in sensitivity and FNR according to the tracer. For patients receiving blue dye only (*n* = 142, three studies), pooled global sensitivity was 90.5% (95%CI: 77.2–96.4%) and pooled FNR was 9.5% (95%CI: 3.6–22.8%). Heterogeneity for this analysis was low as the Higgins’ I^2^ index was 0% (*p* = 0.403). For patients reciving ICG or RC, with or without BD (*n* = 524, seven studies), pooled global sensitivity was 83.5% (95%CI: 61.6%; 94.1%) and pooled FNR was 16.5% (95%CI: 5.9–38.4%). Heterogeneity for this analysis was moderate as the Higgins’ I^2^ index was 70% (*p* = 0.003).

### 3.4. Surgical Complications

Only two studies reported intraoperative complications [26,31]. The first article [26] reported intraoperative complications among 93 patients. They found that one (1.1%) patient suffered from an anaphylactic reaction due to blue dye and six (6.5%) from intraoperative bleeding during lymphadenectomy (and not during the SLN procedure).

The second study [31] mentioned intraoperative and postoperative complications. They noted that eight patients among 268 experienced intraoperative complications (not otherwise specified), however none during the indocyanine green injection or the SLN procedure. Concerning postoperative complications, they reported that 85 (31.7%) women had a postoperative complication within 30 days after surgery. According to the Clavien-Dindo classification, 64 (23.8%) had grade I-II, 19 (7.1%) had grade III, and two (0.7%) had grade IV complication. Six (2.2%) women experienced a serious adverse event after surgery.

### 3.5. Survival and Recurrence

Two articles reported follow-up data [22,33]. The first article [33] described recurrence locations and survival for 52 patients after a median follow-up of 15.6 months. They found that 14 patients (27%) had recurrence and among those, six patients developed recurrence in the pelvic lymph nodes. The authors reported the outcomes of the two patients with false-negative SLN sampling. These patients, who both had serous histology tumors, received adjuvant chemotherapy followed by radiotherapy. Both patients experienced recurrence approximately three months after completing primary therapy. One patient experienced recurrence in the abdomen and died 12 months after diagnosis. The other patient developed a distant and pelvic nodal recurrence and is alive with disease 28 months after diagnosis. One-year disease-free survival was 88% for patients with stage I disease and 44% for patients with stage II+ disease.

The second article [22] reported recurrence locations and survival for 105 patients after a median follow-up of 16 months. They recorded 10 deaths and nine recurrences; among those, two nodals. Of interest, they found that recurrence and death was similar among the group with bilateral SLN sampling plus bilateral pelvic with or without para-aortic lymphadenectomy and the group with bilateral SLN mapping without pelvic lymphadenectomy (in case of a failed mapping in a hemi-pelvis, the patient underwent a side specific pelvic lymphadenectomy, uni- or bi-lateral).

## 4. Discussion

In this meta-analysis of 17 original articles, we assessed the performance of SLN sampling in high risk endometrial cancer in comparison to conventional lymphadenectomy. We found that pooled sensitivity was 88.5% (95%CI: 81.2–93.2%), negative predictive value was 96.0% (95%CI: 93.1–97.7%), and false-negative rate was 11.5% (95%CI: 6.8%; 18.8%). While this technique is already recommended in low and intermediate-risk endometrial cancer, to our knowledge, this is the first meta-analysis of SLN sampling techniques compared to conventional lymphadenectomy specifically in the high risk group. Of interest, we noted heterogeneity in SLN techniques between studies, concerning the tracer and its detection, the injection site, the number of injections, and the surgical approach. Finally, we found a correlation between the number of patients included and the SLN performances. Below, we discuss the feasibility of SLN sampling in high risk endometrial cancer, then its performance, safety, and limits.

### 4.1. SLN Sampling is Also Feasible in High Risk Endometrial Cancer

There is a wide variation in the rate of identification of SLN depending on the studies, with a failure rate ranging from 6.6% to 100% [38] linked in particular to the method of detection and the site of injection of the tracer. In our study, for high risk endometrial cancer, we found a failure rate ranging from 0% to 33%, with a mean value of 11%, and this variation can be explained by the heterogeneity of techniques and the experience of the center, as previously shown in Figure 5. Current scientific literature identifies different factors influencing SLN identification in endometrial cancer, such as the method of detection [39], the site of injection [39], and presence of a gross metastasis in the SLN [33]. On the contrary, some factors are not associated with detection rate such as obesity, histologic type, and tumor grade [39]. We discuss below the different parameters involved in an identification rate, which are available in the selected studies.

The majority of published studies [6,40] have shown that the detection rate of the sentinel node was better if the colorimetric method (using methylene blue or patent blue) was coupled with the injection of a radioactive isotope: Technetium 99 m coupled with rhenium sulfide or albumin, with an overall detection rate of 78% (95%CI: 73–84%) [40]. Recent studies [41,42,43,44], involving a total of 709 patients, have used indocyanine green (ICG) to identify the SLN and it seems that it gives better results with overall detection rates of 94% and bilateral detection rates of 80% [45]. Similarly, a prospective study on 100 patients [46] showed that the use of ICG significantly improved SLN detection over the blue dye, either methylene blue or patent blue, both in overall rate of detected nodes (87% versus 71%, respectively, *p* = 0.005) and in bilateral detection rate (65% versus 43%, respectively, *p* = 0.002). A meta-analysis involving 538 patients, published in 2016 [47], found similar results. Compared to blue dye, ICG SLN sampling has a better overall (odds ratio (OR) 0.27, 95% CI 0.15–0.50, *p* < 0.001) and bilateral (OR 0.27 95% CI 0.19–0.40, *p* < 0.00001) detection rate. When the ICG was compared with Technetium 99 m, there was no difference between the two methods in overall and bilateral detection rates, although these results were based on small series (OR 1.08, 95%CI 0.52–2.26, *p* = 0.83 and OR 1.21, 95%CI 0.80–1.81, *p* = 0.36).

When comparing ICG and blue dye + Technetium-99 m, there was also no difference in the overall detection rate (OR 0.96, 95%CI 0.45–2.02, *p* = 0.91), but a non-significant improvement in the bilateral detection rate (OR 0.37, 95%CI 0.07–2.12, *p* = 0.27). There was no significant difference in the false-negative rate between the ICG and the blue dye (OR 0.26, 95%CI 0.02–3.06, *p* = 0.28) [47]. These results are confirmed by another meta-analysis published in 2016, involving 4915 patients [39], with a higher bilateral detection rate with ICG (75% versus 51%, *p* = 0.008) than with blue dye. The performance of lymphoscintigraphy and the combined use of a radiotracer and dye was associated with higher overall detection rates (86% versus 76%, *p* = 0.016 and 87% versus 78%, *p* = 0.008, respectively), without showing a difference in bilateral and para-aortic SLN detection rates. Therefore, according to the literature, ICG currently appears to be the most effective dye for detecting sentinel lymph nodes in endometrial cancer [39].

Concerning the injection technique, peri-cervical injection is the most common and easiest of the techniques. In addition, its detection rate is the best of the three injection modalities used in endometrial cancer, ranging from 62% to 100% [48]. A meta-analysis of 26 studies using the blue dye [40] showed that peri-cervical injection improved the detection rate (*p* = 0.031), while hysteroscopic injection was associated with a decreased detection rate (*p* = 0.045). Sub-serosal myometrium injection decreased sensitivity (*p* = 0.049) if not combined with other techniques [40]. Rossi et al. [44] found similar results for ICG, with an overall and bilateral detection rate of 82% and 57% after cervical injection versus only 33% and 50% after hysteroscopic injection. In the meta-analysis by Bodurtha Smith et al. [39], peri-cervical injection was associated with a significantly higher bilateral detection rate compared to uterine injection (56% versus 33%, *p* = 0.003). However, peri-cervical injection was also associated with a significantly lower detection rate of para-aortic SLN compared with peri-uterine injection (7% versus 27%, *p* = 0.001). This was not found by Rossi et al. [44] who noted similar rates of detection of para-aortic nodes (71% after peri-cervical injection versus 75% after hysteroscopic injection). Some authors reported hysteroscopic injection, however none were included in this meta-analysis as they did not meet the inclusion criteria. Compared to cervical injection, this technique seems to be as accurate and to have a higher detection rate in the para-aortic area [49,50].

In addition to its greater efficiency, indocyanine green has several advantages over the conventional dual colorimetric and isotopic method [39]. First of all, its safety profile is better: no allergic reactions have been reported in the literature, unlike patent blue. In addition, its injection would be less painful than that of the blue dye [47]. Next, since ICG is highly water-soluble and binds quickly to albumin, it has a propensity for lymphatic tissue [51] and remains more concentrated in the lymph nodes than blue dyes. When injected in the peri-cervical space, the green dye diffuses less towards the rest of the cervix and vagina and at the time of dissection of the retroperitoneal space, it diffuses less outside the lymph nodes, allowing faster and better identification of sentinel lymph nodes and better differentiation from other anatomical structures [47]. In addition, Sinno et al. [52] and Plante et al. [45] showed that the identification of the sentinel node in obese patients was significantly facilitated by the use of ICG compared to blue dye. Finally, a randomised international trial published in 2018 showed that near-infrared detection of ICG is not inferior to BD alone in endometrial cancer [53].

Furthermore, SLN sampling is becoming increasingly more accessible with time. Just a few years ago, this technique was experimental, however today it is widely performed in routine surgical management of endometrial cancer—in particular, for low risk cancer. Indeed, and as previously mentioned, American and French guidelines recommend SLN sampling in low and intermediate-risk early stage endometrial cancer [3,4]. However, this technique must prove its effectiveness in high risk patients.

### 4.2. SLN Sampling Performance in High Risk Endometrial Cancer

Early-stage endometrial cancers at high risk differ from those at low/intermediate risk in terms of the likelihood of lymph node invasion. This prevalence of positive lymph nodes affects the performance of SLN sampling methods. In our study, we found that SLN sampling sensitivity was 88.5% (95%CI: 81.2%–93.2%), negative predictive value was 96.0% (95%CI: 93.1–97.7%), and false-negative rate was 11.5% (95%CI: 6.8%; 18.8%). These results show that SLN sampling is useful in high risk endometrial cancer, especially because the negative predictive value is high. Nevertheless, it must be noted that among the 17 studies selected for the analysis, one appears as an outlier [37] because of the surprisingly high rate of false negative cases (four versus one true positive). A possible explanation is that in this study, the sample size was small (25 patients in total). Accordingly, we found that SLN performances were better in studies with a larger sample size, whether it was for sensitivity, NPV, or FNR. This suggests that a learning curve of this technique could impact its performances. SLN sampling in high risk endometrial cancer could be proposed in expert centers as the number of SLN samplings is higher and is already proposed in routine practice for low-risk patients.

We also investigated whether there was a difference according to the tracer. Only 10 included studies reported information for each tracer. We found that pooled global sensitivity was 90.5% (95%CI: 77.2–96.4%) for patients receiving BD only and 83.5% (95%CI: 61.6%; 94.1%) for patients reciving ICG or RC, with or without BD. Pooled FNR were 9.5% (95%CI: 3.6–22.8%) for BD only and 16.5% (95%CI: 5.9–38.4%) for patients reciving ICG or RC, with or without BD. However, interpretation of these subgroup analyses should be prudent as groups were smaller and heterogeneous. ICG is indeed gaining momentum for SLN detection but, similarly to RC, it is not accessible in all centers performing endometrial cancer surgery and SLN sampling. Subgroup analysis shows that SLN performances are similar for BD only and ICG or RC. However, comparing tracer performances was not the primary aim of our study and therefore, conclusions cannot be reached concerning this point.

Furthermore, SLN sampling allows pathological ultra-staging and thus, detects low-volume metastases that would otherwise be undetected with routine lymphadenectomy evaluations [54]. This technique was not reported for all the studies included in our analysis; consequently, we can hypothesize that its utilization could mitigate the false negative rate. Taken together, these results suggest that SLN sampling performs well in high risk early endometrial cancer and could be valuable in surgical management of these patients, especially since its surgical safety is better than conventional lymphadenectomy.

### 4.3. SLN Sampling Surgical Safety

There are few operative complications related to SLN sampling, unlike the well-known complications of conventional lymphadenectomy, which have been associated in some studies with short and long term operative and post-operative morbidity [12,13,39]. Lymphadenectomy has a complication rate around 26.7% [12]; a significant increase in operative time of more than 30 min (*p* < 0.001) and in the duration of hospitalization (*p* < 0.001); increased blood loss and number of transfusions; an increased number of lesions of the large vessels and nerves; and an increased post-operative complication rate (*p* < 0.05), in particular, lymphoceles, lymphedemas (20%), deep venous thrombosis, or pulmonary embolisms. It has been shown that the risk of post-operative complications increases significantly with the number of nodes harvested, with a 14-node threshold (OR = 2.56, *p* < 0.01) [12].

In addition, it should be remembered that the majority of patients with endometrial cancer are at high surgical risk because of their obesity and associated comorbidities. However, no study has yet investigated the incidence of lymphedema after SLN sampling [39]. The SLN sampling remains of great interest for surgical management of elderly patients. By reducing operative and post-operative morbidity and operative time, this allows lymph node staging in patients where pelvic lymphadenectomy would not have been feasible.

### 4.4. SLN Sampling Contributes to Surgical De-Escalation in Endometrial Cancer

It must be noted that conventional lymphadenectomy has two roles: on one hand, to diagnose if there is lymph node involvement of the disease and on the other hand, to remove the disease spread to the lymphatic system and thus, reduce the tumoral burden. While the latter is still controversial in endometrial cancer, the diagnosis of lymph node involvement determines non-surgical treatments such as radiotherapy. Thus, this diagnostic role has a major impact on treatment and prognosis and this is why today, lymph node involvement is necessary to tailor the therapy for patients with endometrial cancer. Besides, the therapeutic role of lymphadenectomy is still controversial. Indeed, the Cochrane systematic review of 2017 found no evidence that lymphadenectomy decreases the risk of death or disease recurrence compared with no lymphadenectomy in women with presumed stage I disease, and no randomized clinical trials show the impact of lymphadenectomy in women with higher-stage disease and in those at high risk of disease recurrence [55]. Adversely, while the systematic review was based on randomized clinical trials, the literature also contains retrospective analyses showing that pelvic and lombo-aortic lymphadenectomy is associated with good prognosis and better survival for high risk patients, with, in particular, an effect linked to the number of lymph nodes removed [56]. Therefore, the stronger scientific data available at this moment suggest that the role of lymphadenectomy in endometrial cancer remains to diagnose lymph node metastases and to stage the disease, and not to have a therapeutic impact on patients by reducing tumor burden.

That said, SLN sampling is minimally invasive compared to the conventional lymphadenectomy and is able to stage the extent of the disease to lymph nodes with the previously reported performances. This procedure allows surgical de-escalation by avoiding a heavier procedure and obtaining the same diagnosis performance and thus, leading to easier and shorter postoperative recovery.

### 4.5. Limits of the Meta-Analysis

Results of this meta-analysis should be mitigated as some limitations have been identified. First of all, some studies are retrospective and not randomized. Secondly, heterogeneity was present concerning surgical practices and SLN mapping techniques. In addition, results of survival and recurrence should be considered with caution as only two studies were included. Finally, these results were obtained from data coming from expert centers; consequently, their applicability outside these centers should be verified.

## 5. Conclusions

In conclusion, our study supports the hypothesis that SLN sampling is a valuable technique to diagnose lymph node involvement for patients with early stage high risk endometrial cancer in replacement of conventional lymphadenectomy. Consequently, randomized clinical trials are necessary to confirm this hypothesis. In this situation, SLN sampling could lead to surgical de-escalation, while being careful not to undertreat patients.

## Figures and Tables

**Table 1:** Characteristics and results of the studies included in the meta-analysis (2 pages).

Study (Author, Year, Country)	Sentinel Lymph Node Mapping Technique	Surgical Approach	Lymphadenectomy Areas	Total Population Included	Unilateral Detection Rate	Bilateral Detection Rate	True Positive	False Positive	False Negative	True Negative	Sensitivity	Specificity	Positive Predictive Value	Negative Predictive Value	False Negative Rate	Prevalence (N+ Patients)
Baiocchi, 2017, Brazil	BD, cervical injection	Laparoscopy or robotic-assisted laparoscopy	Pelvic with or without para-aortic	75	85%	60%	20	0	2	42	90.90%	100%	100%	95.50%	9.10%	34.38%
Buda, 2018, Italy and Switzerland	BD + RC or ICG, cervical injection	Not available	Pelvic with or without para-aortic	105	98%	80%	29	0	5	71	85.30%	100%	100%	93.40%	14.70%	32.38%
Burke, 1996, United States	BD (isosulfan), subserosal myometrium injection	Laparotomy	Pelvic and para-aortic	15	67%	--	2	0	1	7	66.70%	100%	100%	87.50%	33.30%	30.00%
Ducie, 2017, United States	BD (methylene or isosulfan) or ICG, cervical injection	Not available	Pelvic with or without para-aortic	117	--	--	27	0	1	89	96.40%	100%	100%	98.90%	3.60%	23.93%
Ehrisman, 2016, United States	BD or ICG, cervical injection	Laparoscopy or robotic-assisted laparoscopy	Pelvic with or without para-aortic	36	83%	56%	7	0	2	21	77.80%	100%	100%	91.30%	22.20%	30.00%
Farghali, 2015, Egypt	BD (methylene), subserosal myometrium injection	Laparotomy	Pelvic and para-aortic	93	73%	41%	16	0	1	51	94.10%	100%	100%	98.10%	5.90%	25.00%
Kennard, 2019, United States	ICG with or without BD, cervical injection	Robotic-assisted laparoscopy	Pelvic with or without para-aortic	59	--	--	20	0	3	36	87.00%	100%	100%	92.30%	13.00%	38.98%
Mayoral, 2017, Spain	RC, subserosal myometrium injection	Laparoscopy	Pelvic and para-aortic	11	82%	--	2	0	1	6	66.70%	100%	100%	85.70%	33.30%	33.33%
Naoura, 2015, France	BD and RC, cervical injection	Not available	Pelvic with or without para-aortic	34	88%	59%	10	0	6	14	62.50%	100%	100%	70.00%	37.50%	53.33%
Papadia, 2018, Switzerland	ICG, cervical injection	Laparoscopy	Pelvic and para-aortic	42	100%	90%	9	0	1	32	90.00%	100%	100%	97.00%	10.00%	23.81%
Persson, 2019, Sweden	ICG, cervical injection with or without reinjection	Robotic-assisted laparoscopy	Pelvic and para-aortic	257	100%	95%	52	0	2	203	96.30%	100%	100%	99.00%	3.70%	21.01%
Soliman, 2017, United States	BD, ICG, or RC, alone or combination, cervical injection	Laparoscopy or robotic-assisted laparoscopy or laparotomy	Pelvic and para-aortic	101	89%	58%	19	0	1	70	95.00%	100%	100%	98.60%	5.00%	22.22%
Tanner, 2017, United States	BD (isosulfan) or ICG, cervical injection	Robotic-assisted laparoscopy	Pelvic with or without para-aortic	52	87%	60%	7	0	2	36	77.80%	100%	100%	94.70%	22.20%	20.00%
Torne, 2013, Spain	RC, subserosal myometrium injection	Laparoscopy	Pelvic and para-aortic	74	91%	--	12	0	1	54	92.30%	100%	100%	98.20%	7.70%	19.40%
Touhami, 2017, Canada	BD, ICG, or RC, alone or combination, cervical injection	Laparoscopy or robotic-assisted laparoscopy or laparotomy	Pelvic with or without para-aortic	128	90%	63%	39	0	1	75	97.50%	100%	100%	98.70%	2.50%	34.78%
Wang, 2019, China	ICG, cervical injection	Laparoscopy	Pelvic and para-aortic	98	96%	78%	20	0	2	72	90.90%	100%	100%	97.30%	9.10%	23.40%
Ye, 2019, China	ICG, cervical injection	Laparoscopy	Pelvic and para-aortic	25	100%	72%	1	0	4	20	20.00%	100%	100%	83.30%	80.00%	20.00%

BD = blue dye, ICG = indocyanine green, RC = radiocolloid.
